# Retraction Note: The miR-30a-5p/CLCF1 axis regulates sorafenib resistance and aerobic glycolysis in hepatocellular carcinoma

**DOI:** 10.1038/s41419-025-07492-5

**Published:** 2025-03-07

**Authors:** Zhongqiang Zhang, Xiao Tan, Jing Luo, Hongliang Yao, Zhongzhou Si, Jing-Shan Tong

**Affiliations:** 1 Department of Liver Transplantation, The Second Xiangya Hospital of Central South University, 410011 Changsha, Hunan Province P.R. China; 2 Department of Surgery, University of Pittsburgh School of Medicine, University of Pittsburgh Medical Center Presbyterian Hospital, Pittsburgh, PA 15213 USA; 3 Department of Oncology, Xiangya Hospital, Central South University, 410008 Changsha, Hunan Province P.R. China; 4 Department of General Surgery, The Second Xiangya Hospital of Central South University, 410011 Changsha, Hunan Province P.R. China; 5 Department of Pharmacology and Chemical Biology, University of Pittsburgh School of Medicine, Pittsburgh, PA 15213 USA

Retraction Note to: *Cell Death and Disease* 10.1038/s41419-020-03123-3, published online 23 October 2020

The Editor-in-Chief has retracted this article. An investigation by the National Natural Science Foundation of China Supervision Committee confirmed multiple overlaps in figures between different publications and found these images came from a third party provider. Specifically, two panels from Fig. 2H were found to overlap with three panels from Fig. 5D from another article by different authors [1], Fig. 4D from [2], and Fig. 2K from [3]. Figure 7D was found to overlap with Fig. 6D from [4]. All articles were under consideration at the same time. The Editor has lost confidence in the data and conclusions of this article.

The authors have not responded to correspondence from the publisher regarding this retraction.
